# Penile metastasis in rectal cancer with pathologic complete response after neoadjuvant chemoradiotherapy: The first case report and literature review

**DOI:** 10.1097/MD.0000000000021215

**Published:** 2020-07-17

**Authors:** Taek-Gu Lee, Seung-Myoung Son, Myung Jo Kim, Sang-Jeon Lee

**Affiliations:** a Department of Surgery; b Department of Pathology, Chungbuk National University Hospital, Chungbuk National University College of Medicine, Cheongju, Republic of Korea.

**Keywords:** neoadjuvant chemoradiotherapy, pathologic complete response, penile metastasis, rectal cancer

## Abstract

**Rationale:**

Penile metastasis in rectal cancer is very rare and often originates from prostatic or bladder cancer. The prognosis of penile metastasis is poor and its treatments are more often palliative than curative due to association with disseminated metastases. Pathologic complete response (pCR) in rectal cancer with neoadjuvant chemoradiotherapy (CRT) has been shown to be surrogate marker of favorable long-term outcomes and currently has no report of penile metastasis. Here, we first report isolated penile metastasis in rectal cancer with pCR after neoadjuvant CRT.

**Patient concern:**

The patient was a 74-year-old male with metastasis to the glans penis from rectal cancer diagnosed 9 months after abdominoperineal resection. Physical examination revealed palpable multiple nodules on the glans penis.

**Diagnosis:**

Penile biopsy revealed metastatic carcinoma from the rectal cancer.

**Intervention:**

Chemotherapy was started as soon as possible, because patient suffered urinary discomfort by rapid growing metastatic lesions. He is currently receiving palliative chemotherapy with modified FOLFOX-6 (mFOLFOX-6; oxaliplatin with 5-fluorouracil and folinic acid) plus bevacizumab.

**Outcome:**

The patient is still alive 4 months after diagnosis with markedly decreased metastatic lesions.

**Lesson:**

We propose that although penile metastasis in rectal cancer with pCR after preoperative neoadjuvant CRT is extremely rare, it might help to start early palliative chemotherapy and clinicians should be aware of this possibility.

## 1 Introduction

Despite its abundant vascularization and proximity to the pelvic organ, metastatic involvement of the penis is very rare. Less than 500 cases of penile metastases are reported in literature; genitourinary organs such as prostate and bladder are the most frequently reported sites for the primary cancers.^[1]^ Primary tumors of colorectal cancer are the second common origin of penile metastases. Penile metastases in the colorectal cancers are associated with disseminated disease and poor prognosis.^[2]^ Most of the studies have attempted variable approach for treatment of the penile metastases, such as chemotherapy, total penectomy, and radiotherapy. However, these treatments have been more palliative than curative.^[3–9]^

Recently, many clinicians have performed preoperative neoadjuvant chemoradiotherapy (CRT) for advanced rectal cancer. Neoadjuvant CRT has advantages with respect to oncologic efficacy, safety and anal sphincter preservation. Patients with pathologic complete response (pCR) have especially shown good prognosis.^[10–12]^

Although 6 cases of penile metastasis have been reported for patients who underwent neoadjuvant CRT, there is no case report for patients with pCR. In this case report, we describe for the first time a case of penile metastasis in rectal cancer with pCR after preoperative neoadjuvant CRT. Our report might facilitate decision-making by clinicians with respect to treatment strategies for penile metastasis in rectal cancer.

### 1.1 Consent statement

Written informed consent was obtained from the patient for the publication of this study (Supplemental Digital Content). A copy of the written consent is available for review by the Editor of this journal.

## 2 Case report

**Figure 1:**
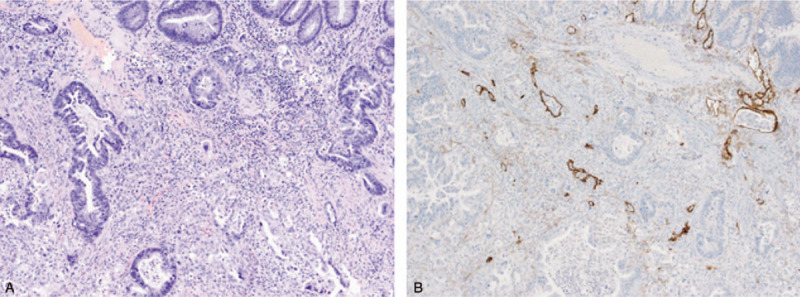
Pathologic findings of the primary tumor. (A) The primary tumor of rectum consisted of moderately differentiated adenocarcinoma with clusters of poorly differentiated tumor cells (H&E. ×100). (B) Immunohistochemistry for D2-40 showed lymphatic invasion of the tumor cells (×100). H&E = hemotoxylin and eosin.

**Figure 2:**
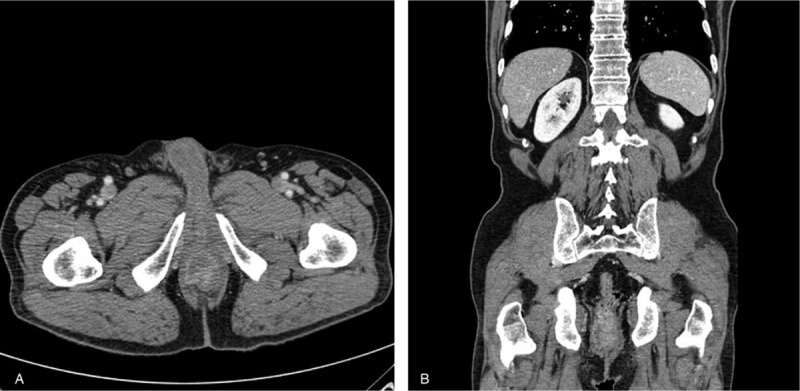
Tumoral infiltration of the rectal cancer on abdominopelvic computed tomography scan. (A) Axial image. (B) Coronal image.

**Figure 3:**
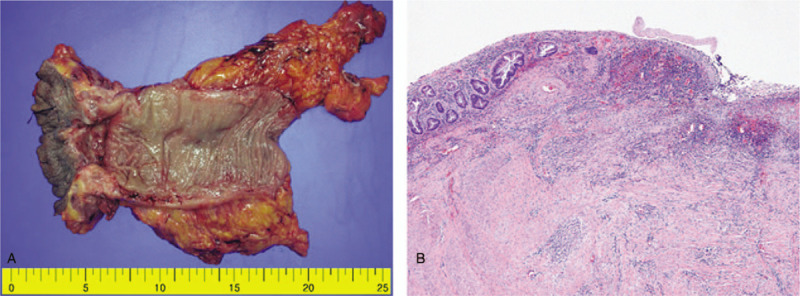
(A) Ulcerative lesion in resected specimen. (B) No residual tumor cells were identified in the resected specimen (×40).

A 74-year-old male presented with bleeding per rectum, constipation, and tenesmus for the past 4 months. Digital rectal examination revealed a luminal encircling mass with irregular surface 5 cm from the anal verge. Colonoscopy showed a large, ulceroinfiltrative mass at 5 cm from the anal verge. Colonoscopic biopsy revealed only hyperplastic crypt epithelium. Transanal incisional biopsy was performed to confirm diagnosis under spinal anesthesia. Histopathologic examination showed that the primary tumor in the rectum consisted of moderate differentiated adenocarcinoma with cluster of poorly differentiated tumor cells (Fig. 1A). Abdominopelvic and chest computed tomography (CT) scan revealed small sized multiple lymph nodes along the superior rectal artery without distant metastasis. The rectal cancer was staged as a cT3N0M0 (Fig. 2). The patient received preoperative neoadjuvant CRT with intensity-modulated radiation therapy (pelvic total dose of 50.4 Gy in 20 fractions) and concomitant oral capecitabine (825 mg/m^2^, twice a day, 5 days per week). Consolidation chemotherapy with capecitabine (1250 mg/m^2^, twice a day, days 1∼14) was performed until the surgery. The patient underwent abdominoperineal resection at 8 weeks after neoadjuvant CRT. The final histopathologic examination report showed in no residual tumor and no metastasis in 12 regional lymph nodes (ypT0N0, Stage 0), and pCR (tumor regression grade 0 by AJCC) (Fig. 3). After the surgery, 5 cycles of capecitabine initiated as adjuvant chemotherapy.

**Figure 4:**
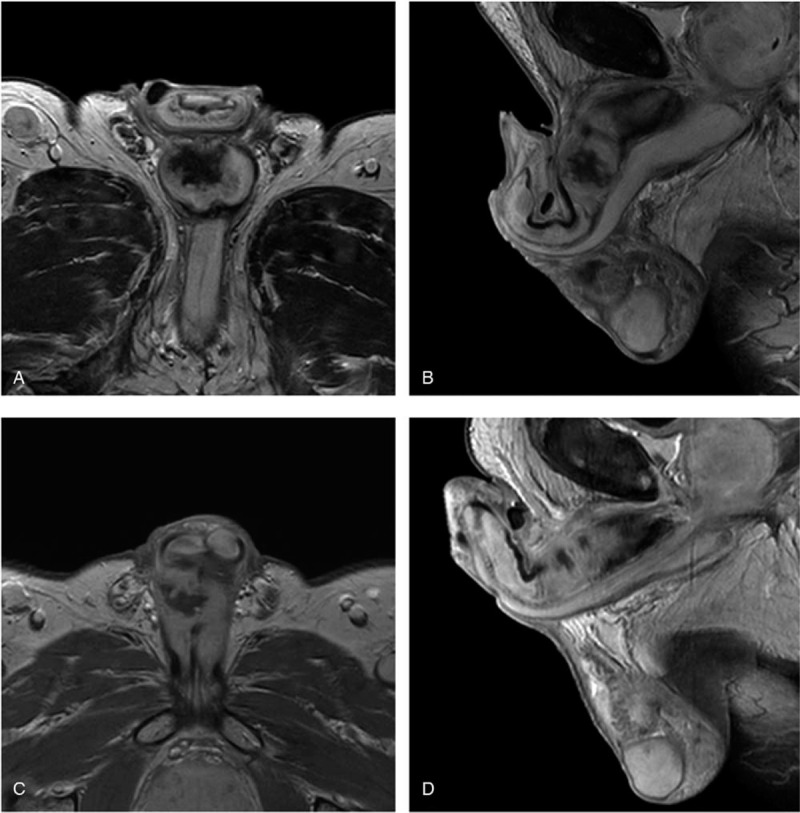
Gadolinium-enhanced fat-suppressed T1-weighted magnetic resonance imaging image showing a low intensity lesion. (A) Axial view before chemotherapy. (B) Sagittal view before chemotherapy. (C) Axial view after chemotherapy. (D) Sagittal view after chemotherapy.

**Figure 5:**
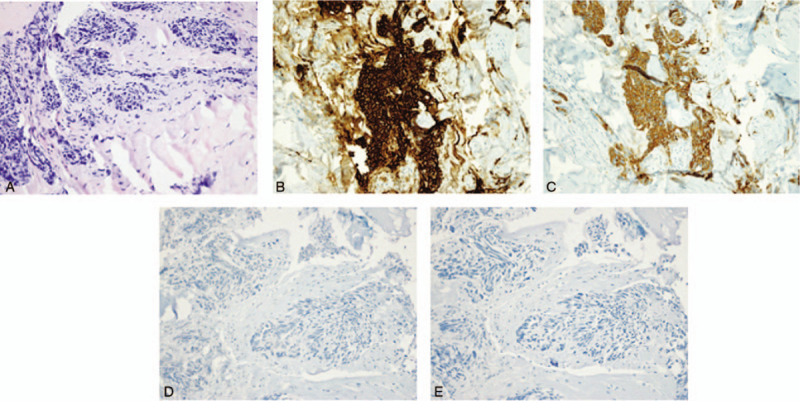
Microscopically, poorly differentiated carcinoma in the penile mass (A) (H&E stain, ×200), which were positive for CK7 (B, ×200) and CK20 (C, ×200), and negative for PSA (D, ×200) and GATA3(E, ×200) by immunohistochemistry. CK = cytokeratin, H&E = hemotoxylin and eosin, PSA = prostate-specific antigen.

Nine months after the surgery, the patient‘s serum carcinoembryonic antigen (CEA) level was elevated (14.94 ng/mL) and a palpable mass was found at the junction of the scrotum and penis. Abdominopelvic CT scan revealed improving state of soft tissue infiltration at the operative site in the perineum. Pelvic magnetic resonance imaging (MRI) showed multiple mass at both penile bulb and both penile corpus cavernosa with metastatic lymph nodes in the right inguinal area (Fig. 4A and B). Incisional biopsy with histopathologic examination showed poorly differentiated carcinoma (Fig. 5A). By immunohistochemistry, tumor cells were positive for CK7 (cytokeratin7), CK20 (cytokeratin20), and CEA, negative for TTF-1 (thyroid transcription factor 1), P63, CDX2 (caudal type homeobox transcription factor 2) (Fig. 5B and C). Additionally, the tumor cells were negative for prostate or genitourinary origin markers including PSA and GATA3 (Fig. 5D and E). Experienced pathologists reviewed the primary tumor slides and found that tumor cells had lymphatic invasion, revealed by positive staining of the D2-40 (Fig. 1B). Metastasis from the rectal adenocarcinoma was confirmed. We summarize the patient‘s event (Table 1). There was no microsatellite instability, as tumor cells were positive for MLH1, MSH2, MSH6, and PMS2 by immunohistochemical evaluation. The patient experienced urinary frequency and difficulty due to very rapidly growing metastatic masses. Therefore, we started palliative chemotherapy with modified FOLFOX-6 (mFOLFOX-6; oxaliplatin with 5-fluorouracil and folinic acid) plus bevacizumab even before confirmation of the genetic profile. The patient is still undergoing palliative chemotherapy. After 8 cycles of palliative chemotherapy, MRI showed markedly decreased mass and symptomatic relief (Fig. 4C and D).

**Table 1:**
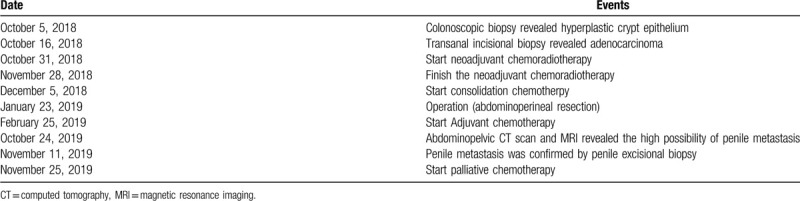
Timeline.

## 3 Discussion

The primary sites of penile metastasis in 75% cases of genitourinary cancers include the prostate, bladder, kidney, and 13% cases of colorectal cancers.^[1]^ Among patients with penile metastasis from the colorectal cancer, two-thirds already have dissemination in the lung, liver, bone, and lymph nodes at diagnosis. In 1 case, penile metastasis was detected about 37 months after primary treatment of colorectal cancer.^[8]^ The common symptoms are urinary frequency, induration, dysuria, priapism, penile pain, and hematuria. The mass usually progresses to involve the corpora cavernosa with extension into the corpus spongiosum, bulb, and neighboring perineal subcutaneous tissue.^[6]^ Penile metastasis should be distinguished from primary penile cancer, chancre, primary syphilis, condyloma acuminate, tuberculosis, Peyronie's disease, and other inflammatory diseases.^[13]^ To confirm the diagnosis of penile metastasis, immunhistochemical staining can be helpful for discrimination of the origin of primary cancer. Metastatic tumor cells from colorectal cancer were shown to be positive for CEA and cytokeratin (CK) 20, and negative for TTF-1 and CK 7.^[7,14]^ In our case, the patient complained of palpable mass with urinary frequency and dysuria without pain. MRI findings showed penile glans, bulb, corpus cavernosa, and right inguinal lymph nodes metastases. The biopsy specimen of penis was positive for CK 20 and CK 7, and negative for PSA, GATA3, TTF-1, and p63. Bayrak et al^[15]^ reported that positivity of CK7 and CK20 was identified in 15.3% of colon adenocarcinoma. In our case, the primary tumor cell had poorly differentiated cluster and invaded lymphatics. Penile lesion also showed poorly differentiated carcinoma. Pathologist disclosed penile metastasis from rectal cancer based on the medical history of the patient and histologic findings. The penis was the first metastatic site from the rectal cancer in 9 months after curative resection.

Various mechanisms have been suggested for penile metastasis such as retrograde venous spread, retrograde lymphatic spread, arterial spread, implantation and secondary to instrumentation or direct extension by Paquin and Roland.^[16]^ The retrograde venous spread seems to be the common route because cancer cells can transport easily through the routes between the dorsal venous system of the penis and the venous plexuses draining the pelvic viscera. This mode well explained the metastasis from prostate, bladder and the rectosigmoid colon cancer. Retrograde lymphatic route is similar to the retrograde venous route but differs from the latter in that there is a lymphatic obstruction instead of a venous block. The anal canal has 2 different lymphatic systems based on the dentate line. Above the dentate line, lymphatic drains cephalad and below, into the inferior rectal lymphatics to the superficial inguinal nodes.^[16,17]^ Lymphatic drainage from the perineal region occurs through the inferior hemorrhoidal vein and internal pudendal vein. Internal pudendal vein communicated with the penis. Inguinal lymph node and penis metastases can occur in the rectal cancer near the dentate line in this route. Arterial occurs due to direct implantation of the circulating tumor cell or tumor embolism. Direct extension is an invasion of the immediate adjacent surrounding organs, and low-lying rectal cancer when located anteriorly can involve the penile root. Implantation or spread by instrumentation can well explain isolated lesion in the corpus spongiosum which is not possible with the route described earlier.^[13,16]^ In our case, the metastatic lesions were located in the corpus cavernosa, penile bulb, and right inguinal lymph nodes. The tumor cells showed lymphatic invasion revealed by positive staining for D2-40 during review of the primary tumor. We speculate that the penile metastasis in this case occurred through the retrograde lymphatic route. The tumor cells transported to the superficial inguinal nodes in the encircling mass just above the dentate line and these nodes communicate with the penis through the inferior rectal lymphatic chain. Multiple lesions in the penis can exclude direct extension.

Approximately 15% to 20% of patients show pCR after neoadjuvant CRT for locally advanced rectal cancer. Recently, pathologic pCR has been shown to be surrogate marker of favorable long-term outcomes such as 5-year overall survival and 5-year recurrence-free survival.^[11]^ A meta-analysis of outcomes following pathologic pCR showed that patients with pCR had 0.7% local recurrence rate and 8.7% distant metastasis rate at a median follow-up of 55.5 months. The 5-year overall survival and 5-year disease-free survival rates were 90.2% and 87.0% in the meta-analysis, respectively.^[12]^ Recently, there has been great interest in the patterns of recurrence in patients with pCR. Fan et al showed that 18 of 195 patients experienced recurrence and the mean recurrence-free survival was 15.1 months. Among those patients, 15 experienced distant metastasis which included 7 lung metastasis, 1 liver metastasis, and 8 metastases in other locations such as the peritoneum, para-aortic lymph node, supraclavicular lymph node, bone, retroperitoneal lymph node, and brain.^[18]^

Until now, only 6 cases of penile metastasis in the rectal cancer after neoadjuvant CRT have been reported.^[3–9]^ Among these, 1 patient did not undergo surgery due to exacerbate multiple pulmonary metastasis and 1 patient received only short-course radiotherapy. Pathologic stages II and III were found in 3 patients each. Four of the 6 cases already had distant metastasis in the lung, liver, and bone and direct invasion of the bladder neck at diagnosis. Duration of the penile metastasis ranged from 4 to 24 months after primary treatment. Survival duration or follow-up of the patients with penile metastasis ranged from 2 to 12 months (Table 2). However, there is no report on penile metastasis in rectal cancer with pCR after neoadjuvant CRT. Total penectomy and CRT have also shown survival from 5 days to 24 months. Patients with penile metastasis consider that disseminated disease and treatment intent was more palliative than adjuvant. In our case, penile metastasis occurred 9 months after treatment despite the patient achieving pCR after neoadjuvant CRT and complete negative resection margin. Systemic palliative chemotherapy with mFOLFOX-6 plus bevacizumab was started as soon as possible in 2 weeks after the penile biopsy, because metastatic lesions grow rapidly. He is still alive 4 months after diagnosis, without urinary discomfort and with markedly decreased metastatic lesions as revealed by MRI.

**Table 2:**
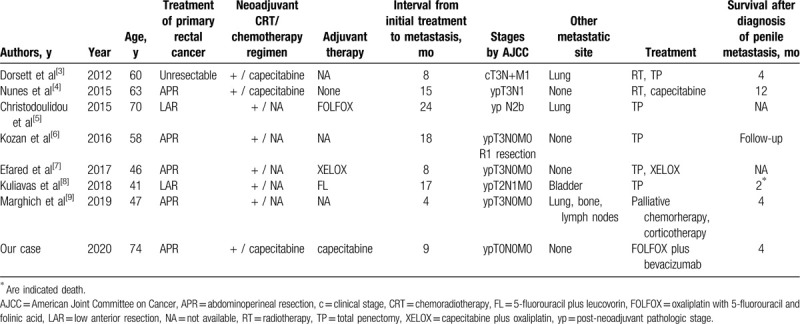
Previous penile metastasis in rectal cancer patients with neoadjuvant chemoradiotherapy.

In conclusion, to our best knowledge, this is the first report on penile metastasis in rectal cancer patient with pCR after neoadjuvant CRT. This case shows that early palliative chemotherapy for penile metastasis might help in achieving better oncologic outcome in progressive growing penile metastases.

## Author contributions

**Conceptualization:** Taek-Gu Lee, Seung-Myoung Son, Myung Jo Kim, Sang-Jeon Lee.

**Data curation:** Taek-Gu Lee, Seung-Myoung Son, Myung Jo Kim.

**Resources:** Sang-Jeon Lee, Taek-Gu Lee, Myung Jo Kim.

**Formal analysis:** Sang-Jeon Lee, Taek-Gu Lee, Seung-Myoung Son

**Supervision:** Sang-Jeon Lee, Seung-Myoung Son, Myung Jo Kim.

**Writing – original draft:** Sang-Jeon Lee, Taek-Gu Lee.

**Writing – review & editing:** Sang-Jeon Lee, Taek-Gu Lee.
